# 2,4-Dichloro-*N*-(3,5-dichloro­phen­yl)benzene­sulfonamide

**DOI:** 10.1107/S160053681104219X

**Published:** 2011-10-22

**Authors:** Vinola Z. Rodrigues, Sabine Foro, B. Thimme Gowda

**Affiliations:** a Department of Chemistry, Mangalore University, Mangalagangotri 574 199, Mangalore, India; b Institute of Materials Science, Darmstadt University of Technology, Petersenstrasse 23, D-64287 Darmstadt, Germany

## Abstract

In the title compound, C_12_H_7_Cl_4_NO_2_S, the N—H bond in the C—SO_2_—NH—C segment is *syn* with respect to the *ortho*-Cl atom of the sulfonyl­benzene ring and one of the *meta*-Cl atoms of the aniline ring. The C—SO_2_—NH—C torsion angle is −93.9 (2)°. The benzene rings are tilted relative to each other by 61.9 (1)°. The crystal structure features inversion-related dimers linked by pairs of N—H⋯O hydrogen bonds.

## Related literature

For the preparation of the title compound, see: Savitha & Gowda (2006 ▶). For hydrogen-bonding modes of sulfonamides, see; Adsmond & Grant (2001 ▶). For studies on the effects of substituents on the structures and other aspects of *N*-(ar­yl)-substituted amides, see: Gowda *et al.* (2000 ▶), on methane­sulfonamides, see: Gowda *et al.* (2007 ▶), on aryl­sulfonamides, see: Gelbrich *et al.* (2007 ▶); Perlovich *et al.* (2006 ▶); Gowda *et al.* (2009 ▶); Shetty & Gowda (2005 ▶) and on *N*-(chloro)-aryl­sulfonamides, see: Gowda *et al.* (2003 ▶).
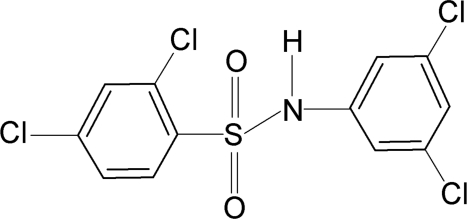


## Experimental

### 

#### Crystal data

C_12_H_7_Cl_4_NO_2_S
*M* *_r_* = 371.05
Triclinic, 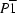

*a* = 8.075 (1) Å
*b* = 9.479 (1) Å
*c* = 10.103 (2) Å
α = 84.90 (2)°
β = 78.49 (1)°
γ = 79.28 (1)°
*V* = 743.46 (19) Å^3^
*Z* = 2
Mo *K*α radiation
μ = 0.93 mm^−1^
*T* = 293 K
0.48 × 0.44 × 0.32 mm

#### Data collection

Oxford Diffraction Xcalibur diffractometer with a Sapphire CCD detector
Absorption correction: multi-scan (*CrysAlis RED*; Oxford Diffraction, 2009 ▶) *T*_min_ = 0.663, *T*_max_ = 0.754
4974 measured reflections
3027 independent reflections
2423 reflections with *I* > 2σ(*I*)
*R*_int_ = 0.014

#### Refinement

*R*[*F* ^2^ > 2σ(*F* ^2^)] = 0.038
*wR*(*F* ^2^) = 0.104
*S* = 1.03
3027 reflections
184 parameters
1 restraint
H atoms treated by a mixture of independent and constrained refinement
Δρ_max_ = 0.36 e Å^−3^
Δρ_min_ = −0.29 e Å^−3^

### 

Data collection: *CrysAlis CCD* (Oxford Diffraction, 2009 ▶); cell refinement: *CrysAlis CCD*; data reduction: *CrysAlis RED* (Oxford Diffraction, 2009 ▶); program(s) used to solve structure: *SHELXS97* (Sheldrick, 2008 ▶); program(s) used to refine structure: *SHELXL97* (Sheldrick, 2008 ▶); molecular graphics: *PLATON* (Spek, 2009 ▶); software used to prepare material for publication: *SHELXL97*.

## Supplementary Material

Crystal structure: contains datablock(s) I, global. DOI: 10.1107/S160053681104219X/rz2649sup1.cif

Structure factors: contains datablock(s) I. DOI: 10.1107/S160053681104219X/rz2649Isup2.hkl

Supplementary material file. DOI: 10.1107/S160053681104219X/rz2649Isup3.cml

Additional supplementary materials:  crystallographic information; 3D view; checkCIF report

## Figures and Tables

**Table 1:** Hydrogen-bond geometry (Å, °)

*D*—H⋯*A*	*D*—H	H⋯*A*	*D*⋯*A*	*D*—H⋯*A*
N1—H1*N*⋯O2^i^	0.82 (2)	2.07 (2)	2.889 (3)	177 (3)

Symmetry code: (i) 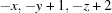.

**Fig. 1.**
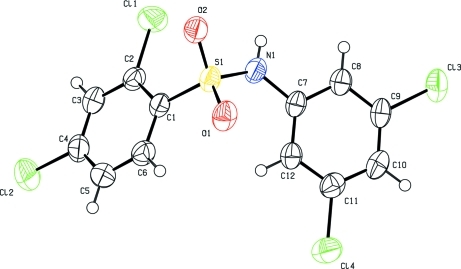
Molecular structure of the title compound, showing displacement ellipsoids drawn at the 50% probability level.

**Fig. 2.**
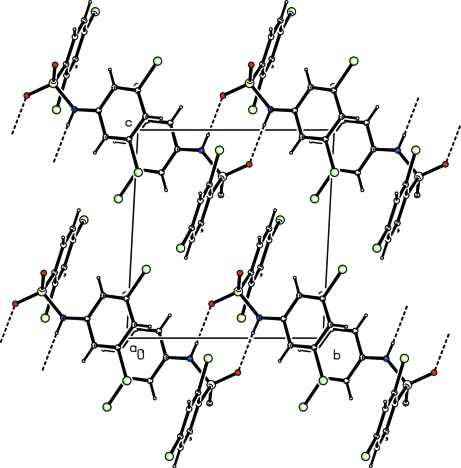
Molecular packing of the title compound with hydrogen bonding shown as dashed lines.

## supplementary crystallographic information

### Comment

The sulfonamide moiety is the constituent of many biologically significant compounds. The hydrogen bonding preferences of sulfonamides have been investigated (Adsmond & Grant, 2001). As part of our studies on the substituent effects on the structures and other aspects of *N*-(aryl)-amides (Gowda *et al.*, 2000), *N*-(aryl)-methanesulfonamides (Gowda *et al.*, 2007), *N*-(aryl)-arylsulfonamides (Gowda *et al.*, 2009; Shetty & Gowda, 2005) and *N*-(chloro)-arylsulfonamides (Gowda *et al.*, 2003), in the present work, the crystal structure of 2,4-dichloro-*N*-(3,5-dichlorophenyl)benzenesulfonamide (I) has been determined (Fig. 1).

In (I), the N—H bond in the C—SO_2_—NH—C segment is *syn* with respect to the *ortho*-Cl atom of the sulfonylbenzene ring and one of the *meta*-Cl atoms of the anilino ring. The molecule is bent at the S atom with the C—SO_2_—NH—C torsion angle of -93.9 (2)°, compared to the value of -48.2 (2)° in 2,4-dichloro-*N*-(3,4-dichlorophenyl)-benzenesulfonamide (II) (Gowda *et al.*, 2009). The sulfonyl and the aniline benzene rings are tilted relative to each other by 61.9 (1)°, compared to the value of 68.9 (1)° in (II). The other bond parameters in (I) are similar to those observed in (II) and other aryl sulfonamides (Perlovich *et al.*, 2006; Gelbrich *et al.*, 2007). In the crystal, intermolecular N–H···O hydrogen bonds (Table 1) link the molecules into dimeric units. Part of the crystal structure is shown in Fig. 2.

### Experimental

A solution of 1,3-dichlorobenzene (10 ml) in chloroform (40 ml) was treated dropwise with chlorosulfonic acid (25 ml) at 273 K. After the initial evolution of hydrogen chloride subsided, the reaction mixture was brought to room temperature and poured into crushed ice in a beaker. The chloroform layer was separated, washed with cold water and allowed to evaporate slowly. The residual 2,4-dichlorobenzenesulfonylchloride was treated with 3,5-dichloroaniline in the stoichiometric ratio and boiled for ten minutes. The reaction mixture was then cooled to room temperature and added to ice cold water (100 ml). The resultant solid 2,4-dichloro-*N*-(3,5-dichlorophenyl)benzenesulfonamide was filtered under suction and washed thoroughly with cold water. It was then recrystallized to constant melting point from dilute ethanol. The purity of the compound was checked and characterized by recording its infrared and NMR spectra (Savitha & Gowda, 2006). Prism like light pink single crystals used in X-ray diffraction studies were grown in ethanolic solution by slow evaporation of the solvent at room temperature.

### Refinement

The H atoms of the NH group was located in a difference Fourier map and refined with the N—H distance restrained to 0.86 (2) Å and with *U*_iso_(H) = 1.2*U*_eq_(N). All other H atoms were positioned with idealized geometry using a riding model with C—H = 0.93 Å,and refined with *U*_iso_(H) values set at 1.2*U*_eq_(C).

### Crystal data

**Table:**

C_12_H_7_Cl_4_NO_2_S	*Z* = 2
*M**_r_* = 371.05	*F*(000) = 372
Triclinic, *P*1	*D*_x_ = 1.657 Mg m^−^^3^
Hall symbol: -P 1	Mo *K*α radiation, λ = 0.71073 Å
*a* = 8.075 (1) Å	Cell parameters from 2149 reflections
*b* = 9.479 (1) Å	θ = 2.6–27.7°
*c* = 10.103 (2) Å	µ = 0.93 mm^−^^1^
α = 84.90 (2)°	*T* = 293 K
β = 78.49 (1)°	Prism, light pink
γ = 79.28 (1)°	0.48 × 0.44 × 0.32 mm
*V* = 743.46 (19) Å^3^	

### Data collection

**Table:**

Oxford Diffraction Xcalibur diffractometer with a Sapphire CCD detector	3027 independent reflections
Radiation source: fine-focus sealed tube	2423 reflections with *I* > 2σ(*I*)
graphite	*R*_int_ = 0.014
Rotation method data acquisition using ω scans	θ_max_ = 26.4°, θ_min_ = 2.6°
Absorption correction: multi-scan (*CrysAlis RED*; Oxford Diffraction, 2009)	*h* = −10→10
*T*_min_ = 0.663, *T*_max_ = 0.754	*k* = −11→11
4974 measured reflections	*l* = −12→12

### Refinement

**Table:**

Refinement on *F*^2^	Primary atom site location: structure-invariant direct methods
Least-squares matrix: full	Secondary atom site location: difference Fourier map
*R*[*F*^2^ > 2σ(*F*^2^)] = 0.038	Hydrogen site location: inferred from neighbouring sites
*wR*(*F*^2^) = 0.104	H atoms treated by a mixture of independent and constrained refinement
*S* = 1.03	*w* = 1/[σ^2^(*F*_o_^2^) + (0.0494*P*)^2^ + 0.2632*P*] where *P* = (*F*_o_^2^ + 2*F*_c_^2^)/3
3027 reflections	(Δ/σ)_max_ = 0.001
184 parameters	Δρ_max_ = 0.36 e Å^−^^3^
1 restraint	Δρ_min_ = −0.29 e Å^−^^3^

### Special details

**Table:**

Geometry. All e.s.d.'s (except the e.s.d. in the dihedral angle between two l.s. planes) are estimated using the full covariance matrix. The cell e.s.d.'s are taken into account individually in the estimation of e.s.d.'s in distances, angles and torsion angles; correlations between e.s.d.'s in cell parameters are only used when they are defined by crystal symmetry. An approximate (isotropic) treatment of cell e.s.d.'s is used for estimating e.s.d.'s involving l.s. planes.
Refinement. Refinement of *F*^2^ against ALL reflections. The weighted *R*-factor *wR* and goodness of fit *S* are based on *F*^2^, conventional *R*-factors *R* are based on *F*, with *F* set to zero for negative *F*^2^. The threshold expression of *F*^2^ > σ(*F*^2^) is used only for calculating *R*-factors(gt) *etc*. and is not relevant to the choice of reflections for refinement. *R*-factors based on *F*^2^ are statistically about twice as large as those based on *F*, and *R*- factors based on ALL data will be even larger.

### Fractional atomic coordinates and isotropic or equivalent isotropic displacement parameters (Å^2^)

**Table:**

	*x*	*y*	*z*	*U*_iso_*/*U*_eq_	
C1	0.2231 (3)	0.3850 (2)	0.6848 (2)	0.0383 (5)	
C2	0.3719 (3)	0.3794 (3)	0.7381 (2)	0.0401 (5)	
C3	0.5311 (3)	0.3381 (3)	0.6599 (3)	0.0454 (6)	
H3	0.6298	0.3351	0.6951	0.055*	
C4	0.5432 (3)	0.3010 (3)	0.5291 (3)	0.0481 (6)	
C5	0.3988 (3)	0.3048 (3)	0.4738 (3)	0.0520 (6)	
H5	0.4086	0.2792	0.3855	0.062*	
C6	0.2400 (3)	0.3475 (3)	0.5529 (2)	0.0466 (6)	
H6	0.1419	0.3512	0.5167	0.056*	
C7	−0.0988 (3)	0.2062 (2)	0.9076 (2)	0.0397 (5)	
C8	−0.1821 (3)	0.1626 (3)	1.0339 (3)	0.0431 (5)	
H8	−0.1863	0.2132	1.1096	0.052*	
C9	−0.2587 (3)	0.0423 (3)	1.0452 (3)	0.0442 (6)	
C10	−0.2563 (3)	−0.0345 (3)	0.9350 (3)	0.0482 (6)	
H10	−0.3092	−0.1149	0.9440	0.058*	
C11	−0.1723 (3)	0.0125 (3)	0.8107 (3)	0.0455 (6)	
C12	−0.0919 (3)	0.1312 (3)	0.7950 (3)	0.0436 (5)	
H12	−0.0345	0.1599	0.7105	0.052*	
N1	−0.0178 (3)	0.3284 (2)	0.9021 (2)	0.0470 (5)	
H1N	−0.017 (4)	0.358 (3)	0.975 (2)	0.056*	
O1	−0.1015 (2)	0.4407 (2)	0.68767 (18)	0.0528 (5)	
O2	0.0147 (2)	0.57618 (18)	0.83484 (17)	0.0488 (4)	
Cl1	0.36340 (9)	0.42083 (9)	0.90298 (7)	0.0630 (2)	
Cl2	0.74509 (9)	0.25049 (9)	0.43243 (8)	0.0707 (2)	
Cl3	−0.36154 (9)	−0.01334 (8)	1.20398 (7)	0.0631 (2)	
Cl4	−0.16598 (10)	−0.08094 (8)	0.66939 (8)	0.0692 (2)	
S1	0.01475 (7)	0.44418 (6)	0.77605 (6)	0.04076 (17)	

### Atomic displacement parameters (Å^2^)

**Table:**

	*U*^11^	*U*^22^	*U*^33^	*U*^12^	*U*^13^	*U*^23^
C1	0.0362 (12)	0.0411 (12)	0.0409 (12)	−0.0147 (10)	−0.0093 (9)	0.0030 (10)
C2	0.0375 (12)	0.0447 (13)	0.0423 (13)	−0.0134 (10)	−0.0122 (10)	−0.0015 (10)
C3	0.0359 (12)	0.0481 (14)	0.0548 (15)	−0.0136 (11)	−0.0101 (11)	0.0010 (11)
C4	0.0428 (14)	0.0466 (14)	0.0531 (15)	−0.0151 (11)	0.0007 (11)	0.0003 (11)
C5	0.0567 (16)	0.0606 (16)	0.0418 (14)	−0.0193 (13)	−0.0069 (12)	−0.0047 (12)
C6	0.0455 (14)	0.0566 (15)	0.0436 (14)	−0.0189 (12)	−0.0148 (11)	0.0007 (11)
C7	0.0274 (11)	0.0447 (13)	0.0487 (13)	−0.0103 (10)	−0.0098 (9)	0.0027 (10)
C8	0.0339 (12)	0.0466 (13)	0.0491 (14)	−0.0096 (10)	−0.0080 (10)	0.0016 (11)
C9	0.0288 (11)	0.0477 (14)	0.0547 (14)	−0.0092 (10)	−0.0071 (10)	0.0089 (11)
C10	0.0354 (13)	0.0437 (13)	0.0681 (17)	−0.0129 (11)	−0.0125 (12)	0.0020 (12)
C11	0.0335 (12)	0.0459 (13)	0.0584 (15)	−0.0044 (10)	−0.0117 (11)	−0.0081 (12)
C12	0.0311 (12)	0.0501 (14)	0.0495 (14)	−0.0098 (10)	−0.0057 (10)	−0.0004 (11)
N1	0.0489 (12)	0.0557 (12)	0.0430 (11)	−0.0264 (10)	−0.0104 (10)	0.0028 (10)
O1	0.0390 (9)	0.0673 (12)	0.0570 (11)	−0.0153 (8)	−0.0199 (8)	0.0081 (9)
O2	0.0475 (10)	0.0473 (10)	0.0518 (10)	−0.0135 (8)	−0.0059 (8)	−0.0004 (8)
Cl1	0.0509 (4)	0.0957 (6)	0.0500 (4)	−0.0160 (4)	−0.0187 (3)	−0.0163 (4)
Cl2	0.0511 (4)	0.0808 (5)	0.0728 (5)	−0.0128 (4)	0.0116 (3)	−0.0131 (4)
Cl3	0.0566 (4)	0.0687 (5)	0.0631 (4)	−0.0273 (4)	−0.0004 (3)	0.0123 (3)
Cl4	0.0673 (5)	0.0699 (5)	0.0748 (5)	−0.0190 (4)	−0.0071 (4)	−0.0263 (4)
S1	0.0344 (3)	0.0473 (3)	0.0433 (3)	−0.0139 (3)	−0.0101 (2)	0.0040 (3)

### Geometric parameters (Å, °)

**Table:**

C1—C6	1.383 (3)	C7—N1	1.424 (3)
C1—C2	1.402 (3)	C8—C9	1.381 (3)
C1—S1	1.765 (2)	C8—H8	0.9300
C2—C3	1.376 (3)	C9—C10	1.378 (4)
C2—Cl1	1.731 (2)	C9—Cl3	1.738 (2)
C3—C4	1.378 (4)	C10—C11	1.381 (4)
C3—H3	0.9300	C10—H10	0.9300
C4—C5	1.383 (4)	C11—C12	1.383 (3)
C4—Cl2	1.731 (3)	C11—Cl4	1.733 (3)
C5—C6	1.379 (4)	C12—H12	0.9300
C5—H5	0.9300	N1—S1	1.618 (2)
C6—H6	0.9300	N1—H1N	0.815 (16)
C7—C12	1.381 (3)	O1—S1	1.4254 (17)
C7—C8	1.386 (3)	O2—S1	1.4319 (18)
			
C6—C1—C2	118.7 (2)	C7—C8—H8	120.7
C6—C1—S1	118.24 (17)	C10—C9—C8	122.1 (2)
C2—C1—S1	123.06 (18)	C10—C9—Cl3	119.25 (18)
C3—C2—C1	120.3 (2)	C8—C9—Cl3	118.6 (2)
C3—C2—Cl1	117.68 (17)	C9—C10—C11	117.6 (2)
C1—C2—Cl1	121.97 (18)	C9—C10—H10	121.2
C2—C3—C4	119.5 (2)	C11—C10—H10	121.2
C2—C3—H3	120.3	C10—C11—C12	122.3 (2)
C4—C3—H3	120.3	C10—C11—Cl4	119.07 (19)
C3—C4—C5	121.6 (2)	C12—C11—Cl4	118.7 (2)
C3—C4—Cl2	118.57 (19)	C7—C12—C11	118.5 (2)
C5—C4—Cl2	119.9 (2)	C7—C12—H12	120.8
C6—C5—C4	118.4 (2)	C11—C12—H12	120.8
C6—C5—H5	120.8	C7—N1—S1	127.32 (17)
C4—C5—H5	120.8	C7—N1—H1N	115 (2)
C5—C6—C1	121.6 (2)	S1—N1—H1N	114 (2)
C5—C6—H6	119.2	O1—S1—O2	119.41 (11)
C1—C6—H6	119.2	O1—S1—N1	109.48 (10)
C12—C7—C8	120.9 (2)	O2—S1—N1	105.64 (11)
C12—C7—N1	122.7 (2)	O1—S1—C1	106.86 (11)
C8—C7—N1	116.4 (2)	O2—S1—C1	108.24 (10)
C9—C8—C7	118.7 (2)	N1—S1—C1	106.57 (11)
C9—C8—H8	120.7		
			
C6—C1—C2—C3	0.3 (3)	Cl3—C9—C10—C11	−179.56 (18)
S1—C1—C2—C3	−177.25 (18)	C9—C10—C11—C12	0.4 (4)
C6—C1—C2—Cl1	−178.67 (18)	C9—C10—C11—Cl4	179.96 (17)
S1—C1—C2—Cl1	3.7 (3)	C8—C7—C12—C11	0.9 (3)
C1—C2—C3—C4	−0.5 (4)	N1—C7—C12—C11	179.0 (2)
Cl1—C2—C3—C4	178.53 (19)	C10—C11—C12—C7	−1.1 (4)
C2—C3—C4—C5	0.2 (4)	Cl4—C11—C12—C7	179.34 (17)
C2—C3—C4—Cl2	179.58 (18)	C12—C7—N1—S1	30.7 (3)
C3—C4—C5—C6	0.3 (4)	C8—C7—N1—S1	−151.21 (19)
Cl2—C4—C5—C6	−179.1 (2)	C7—N1—S1—O1	21.3 (3)
C4—C5—C6—C1	−0.5 (4)	C7—N1—S1—O2	151.1 (2)
C2—C1—C6—C5	0.2 (4)	C7—N1—S1—C1	−93.9 (2)
S1—C1—C6—C5	177.9 (2)	C6—C1—S1—O1	0.0 (2)
C12—C7—C8—C9	−0.1 (3)	C2—C1—S1—O1	177.56 (19)
N1—C7—C8—C9	−178.3 (2)	C6—C1—S1—O2	−129.83 (19)
C7—C8—C9—C10	−0.6 (4)	C2—C1—S1—O2	47.8 (2)
C7—C8—C9—Cl3	179.45 (17)	C6—C1—S1—N1	116.95 (19)
C8—C9—C10—C11	0.5 (4)	C2—C1—S1—N1	−65.5 (2)

### Hydrogen-bond geometry (Å, °)

**Table:**

*D*—H···*A*	*D*—H	H···*A*	*D*···*A*	*D*—H···*A*
N1—H1N···O2^i^	0.82 (2)	2.07 (2)	2.889 (3)	177 (3)

Symmetry codes: (i) −*x*, −*y*+1, −*z*+2.

## References

[bb1] Adsmond, D. A. & Grant, D. J. W. (2001). *J. Pharm. Sci.* **90**, 2058–2077. 10.1002/jps.1157 11745765

[bb2] Gelbrich, T., Hursthouse, M. B. & Threlfall, T. L. (2007). *Acta Cryst.* B**63**, 621–632. 10.1107/S010876810701395X 17641433

[bb3] Gowda, B. T., D’Souza, J. D. & Kumar, B. H. A. (2003). *Z. Naturforsch. Teil A*, **58**, 51–56.

[bb4] Gowda, B. T., Foro, S. & Fuess, H. (2007). *Acta Cryst.* E**63**, o2337.

[bb5] Gowda, B. T., Foro, S., Nirmala, P. G. & Fuess, H. (2009). *Acta Cryst.* E**65**, o1940. 10.1107/S1600536809027883 PMC2977279 21583622

[bb6] Gowda, B. T., Svoboda, I. & Fuess, H. (2000). *Z. Naturforsch. Teil A* **55**, 779–790.

[bb7] Oxford Diffraction (2009). *CrysAlis CCD* and *CrysAlis RED* Oxford Diffraction Ltd, Yarnton, England.

[bb8] Perlovich, G. L., Tkachev, V. V., Schaper, K.-J. & Raevsky, O. A. (2006). *Acta Cryst.* E**62**, o780–o782.

[bb9] Savitha, M. B. & Gowda, B. T. (2006). *Z. Naturforsch. Teil A*, **61**, 600–606.

[bb10] Sheldrick, G. M. (2008). *Acta Cryst.* A**64**, 112–122. 10.1107/S0108767307043930 18156677

[bb11] Shetty, M. & Gowda, B. T. (2005). *Z. Naturforsch. Teil A*, **60**, 113–120.

[bb12] Spek, A. L. (2009). *Acta Cryst.* D**65**, 148–155. 10.1107/S090744490804362X PMC2631630 19171970

